# Effects and Assessment of the Optic Pathway After Management with Stereotactic Radiosurgery for Intracranial Tumors: A Comprehensive Literature Review

**DOI:** 10.7759/cureus.43538

**Published:** 2023-08-15

**Authors:** Monica Aguirre Maqueda, Lilian Zavala Romero, Rodrigo Monroy Córdoba, Juan Marcos Meraz Soto, Jorge Alejandro Torres-Ríos, Daniel Ballesteros Herrera, Alejandro Rodríguez Camacho, Sergio Moreno Jiménez

**Affiliations:** 1 Neuro Radiosurgery Department, National Institute of Neurology and Neurosurgery, Mexico City, MEX; 2 Ophthalmology Department, Association to Prevent Blindness (APEC), Mexico City, MEX; 3 Neuro Radiosurgery Department, American British Cowdray Medical Center, Mexico City, MEX

**Keywords:** radiation-induced optic neuropathy, visual pathway, intracranial tumors, radiotherapy, stereotactic radiosurgery

## Abstract

Intracranial tumors are treated through a minimally invasive procedure called stereotactic radiosurgery (SRS), which uses precisely targeted radiation beams. When SRS is used to treat tumors in or near the optic pathway, which is responsible for transmitting visual information from the eyes to the brain, it is essential to assess the effects of treatment on visual function. The optic pathway is considered relatively radiation-sensitive, and high doses of radiation can lead to visual impairment or loss. Various methods can be used to assess the effects of SRS on the optic pathway, including visual acuity testing, visual field testing, and imaging studies. These assessments can be performed before and after treatment to track changes in visual function and detect potential complications or side effects. Assessing the optic pathway after management with SRS for intracranial tumors is essential to the treatment process to ensure that patients receive the best possible outcomes while minimizing the risk of complications. Close collaboration between the multidisciplinary team is often necessary to optimize treatment planning and monitoring of treatment response. In this review, we conducted an extensive analysis of the effects of radiation in patients with intracranial tumors after receiving radiotherapy.

## Introduction and background

Stereotactic radiosurgery (SRS) utilizes externally generated ionizing radiation to inactivate or eradicate defined target(s) in the head and spine without making an incision. It treats primary brain tumors, such as meningiomas, vestibular schwannomas, sellar and suprasellar lesions; brain metastases; and arteriovenous malformations. The latest developments have been in its modalities, and it remains a powerful, minimally invasive instrument that offers additional options for intervention in selected patients. SRS/fractionated stereotactic radiotherapy (FSRT) is the treatment of choice if surgery is considered too risky, as an adjuvant treatment to surgery, when complete surgical resection is not possible, or with residual or recurrent disease [1].

Focusing on those tumor lesions, we can distinguish three entities that involve the optic pathway: tumors that directly compromise structures of the eye, orbit, or optic pathway (i.e., melanoma, glioblastoma of the optic nerve); tumors that compress the optic structures and may themselves cause visual symptoms (i.e., sellar lesions that compress the chiasm); and peri-optic lesions (primary or metastatic brain tumors) that are within a few millimeters of the optic apparatus that may also lead to optic structures becoming “organs at risk” (OAR) after surgical or radiological management. 

The single and multi-fraction SRS dose tolerances of the optic pathways have been described, and current radiosurgery protocols are becoming increasingly precise. However, it remains a challenge to balance a therapy capable of reducing tumor volume and effects while protecting the visual pathway and other structures that are characteristically more susceptible to radiation damage. The accurate patient alignment and sharp dose gradients of stereotactic techniques minimize the optic apparatus dose exposure [2]. This study aims to review the literature on radiosurgery's effects, risks, and harm prevention on intracranial tumors related to the visual pathway. More and more patients with intracranial tumors are being treated with radiosurgery, so knowledge of the radiobiological mechanisms and their clinical manifestations will prevent the diagnosis of "optic damage secondary to radiation" from being overestimated and confused with other causes of visual deficits more common in these patients. We will also review those causes of visual impairment that the evaluator should consider in the patient's post-irradiation follow-up to show a broader perspective of the differential diagnoses of optical damage secondary to radiation. This is important because each scenario will have a different prognosis and management.

## Review

Material and methods 

Our eligibility criteria were included if mainly or part of it shows information about the effects of SRS on the visual pathway. We excluded articles whose full text is not in English and articles not found. Search strategy In the PubMed search engine consisted of the item ((optic pathway) OR (visual pathway) AND (stereotactic radiosurgery) OR (radiosurgery). The database coverage was established from 1945 to 2023. In the Google Scholar search engine, the search was done by requesting articles with the terms “optic pathway,” “visual pathway,” “radiosurgery,” and “stereotactic radiosurgery” somewhere in the report. Only articles published from 1982 to date were requested. Primarily 25,555 results were obtained from the PubMed search and 17,500 results from Google Scholar. To decrease bias, the results were hierarchically organized utilizing the relevance function on Google Scholar, and the first 200 articles were included in the initial screening. Repeated results within the searches were later eliminated. The first 200 articles were included in the initial screening. Repeated results within the searches were later eliminated. The results were arranged by relevance according to the search engine. The abstract of the first 200 results in both searches was read by four reviewers independently. The abstracts were carefully read by reading the title and abstract. All articles were evaluated to assess relevance for this review. Four researchers were assigned to the task independently. After the initial screening, they carefully read the complete abstracts and assessed the entire articles considering only the most relevant according to their suitability and appropriateness for this review. All selected articles from four researchers were critically evaluated by a fifth independent researcher who also established the most relevant writings according to the same criteria. Articles selected by all five researchers independently in consensus were directly included in the primary analysis. Articles with methodological inconsistencies, interventions other than radiosurgery, and non-conclusive results were eliminated. Later, a final agreement on the selected articles was agreed upon by researchers.

The visual pathway consists of the path from the retina to the cortex. The retina has five types of neurons: photoreceptors, bipolar cells, ganglion cells, horizontal cells, and amacrine cells [3]. A direct three-neuron chain-photoreceptor cell to bipolar cell to ganglion cell is the primary route of information flow from photoreceptors to the optic nerve. The much larger axons of the ganglion cells form the optic nerve and carry information about retinal stimulation to the rest of the CNS. The optic nerves are phylogenetically an evagination of the brain. About four millimeters nasal to the central posterior pole of the globe, the nerve fibers of the inner ocular superficial layer converge and pierce the outer retina, the choroid, and the lamina cribrosa, where they form the optic nerve head and the intraocular segment of the optic nerve [4]. These pass into the skull from the orbits via the optic canals about five millimeters long and 3-4 mm wide [4,5]. The course of the optic nerve can be subdivided into an intraocular, an intraorbital, a canalicular, and an intracranial segment [4]. The optic nerves enter the middle cranial fossa, and at a point anterior to the infundibulum of the pituitary gland, the medial fibers decussate to form the optic chiasm on the base of the brain [5,6]. It lies immediately rostral to the tuber cinereum of the hypothalamus and between the terminating internal carotid arteries. In the chiasm, axons derived from the nasal halves of the two retinae decussate pass into the contralateral optic tract, while those from the temporal hemiretina remain ipsilateral [6]. Once past the chiasm, the ganglion cell axons on each side form the optic tract [3]. They diverge away from the chiasm and pass around the cerebral peduncle to terminate mainly in the lateral geniculate nucleus of the thalamus [6].

**Figure 1:**
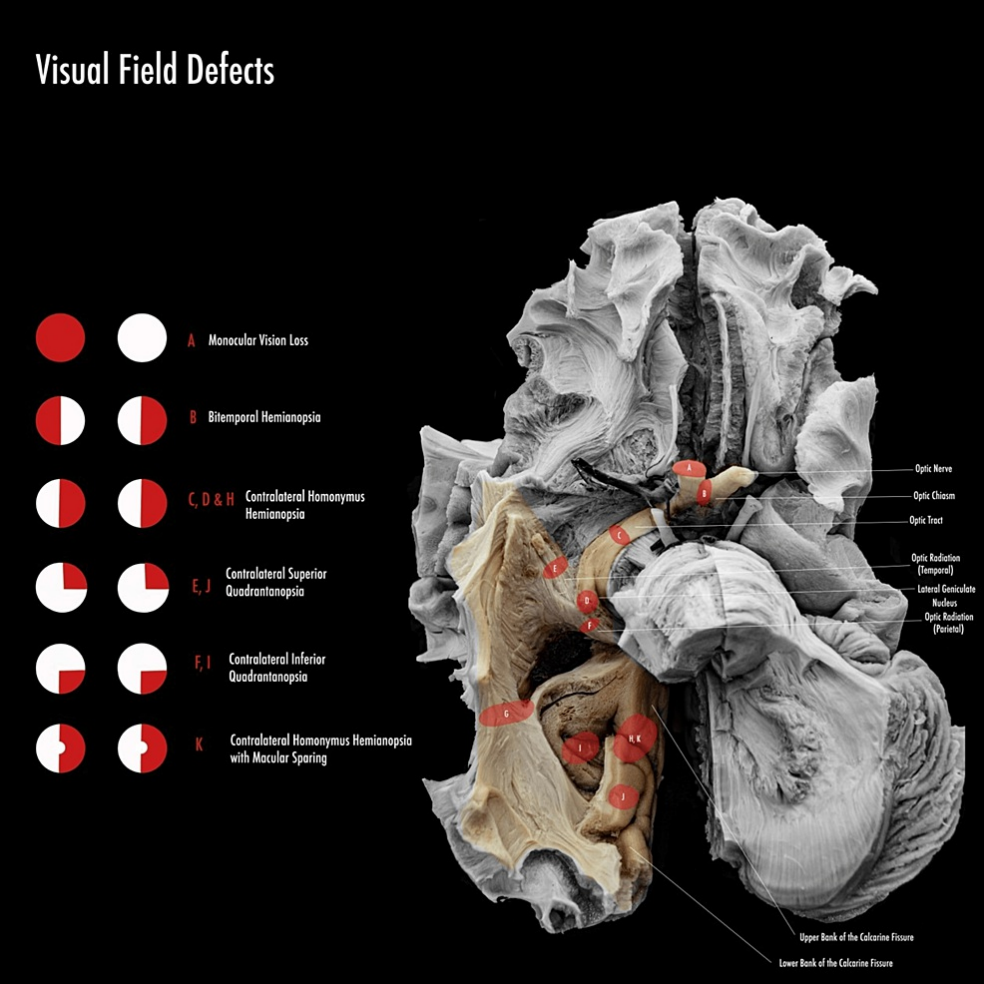
Anatomic representation of the visual pathway and location of the visual field defects.

A few fibers leave the optic nerve before reaching the lateral geniculate nucleus to terminate in the pretectal area and the superior colliculus. These fibers are involved in the mediation of the pupillary light reflex. Third-order thalamocortical neurons from the lateral geniculate nucleus project through the retro-lenticular part of the internal capsule and form the optic radiation, or geniculocalcarine tracts, which terminates in the primary visual cortex of the occipital lobe [5,6]. The inferior fibers contain information about the superior visual field and initially pass anteriorly as a Meyer loop, lateral to the anterior portion of the temporal horn of the lateral ventricle, then course through the temporal lobes to terminate in the primary visual cortex below the calcarine fissure in the medial surface of the occipital lobe [5]. The superior tracts represent the lower part of the visual field. They travel through the parietal lobe, form part of the wall of the superior aspect of the lateral ventricle, and terminate in the visual cortex above the calcarine sulcus [5,6]. The primary visual cortex, also called Brodmann’s area 17 or V1, is located on the medial surfaces of the occipital lobes above and below the calcarine fissures [3,5]. The rest of the occipital lobe constitutes the visual association cortex. It concerns the interpretation of visual images, recognition, depth perception, and color vision [6]. A second primary target of the ganglion cell axons is a collection of neurons between the thalamus and the midbrain in a region known as the pretectum, which is particularly important as the coordinating center for the pupillary light reflex [3]. There is a precise point-to-point relationship between the retina and the visual cortex. Objects in either half of the visual field produce images upon the nasal hemiretina of the ipsilateral eye and the temporal hemiretina of the contralateral eye (Figure 1) [6].

Intracranial tumors associated with visual pathway location

Retinoblastoma

The most common first signs are leukocoria, strabismus, and a mass protruding from the eye; the less common clinical signs include severe ocular inflammations, hyphema, and glaucoma. Visual loss was also reported in some cases. The diagnosis may be difficult when the ocular fundus cannot be adequately examined because of cataracts or massive retinal detachment [7,8]. In a review of six adult patients, there were characteristic signs of fundus appearance, including unilateral diseases, white mass originating from the retina with vitreous seeds, tumor-associated feeding vessels, exudative retinal detachment, subretinal discrete white-yellow deposits, and uncommon calcification [9].

Hemangioma

Circumscribed choroidal hemangioma appears as a discrete, smooth, homogenous surface, round or oval, orange-reddish mass located in the posterior pole. Subfoveal tumors produce hyperopic shifts secondary to the anterior displacement of the retina. Tumors may remain asymptomatic until adulthood when they present with gradual or sudden onset of blurred vision secondary to exudative retinal detachment [10]. Visual acuity can vary from 20/20 to hand motions. Patients also present metamorphopsia, field defects, floaters, macular edema, retinal and subretinal exudates, and, in long-standing cases, epiretinal membrane and choroidal neovascular membrane [11].

Diffuse choroidal hemangioma is usually diagnosed due to visual impairment caused by hyperopic amblyopia. The pupil shows a brilliant red reflex (tomato catsup fundus) in the involved eye. Ophthalmoscopy reveals a diffuse red-orange thickening of the posterior choroid, mainly seen in the macular area, that may cause retinal defocus, refractive error, and hyperopic amblyopia. Cystoid degeneration in the overlying retina over the tumor surface with RPE disruption commonly occurs. Asymmetry of the optic disc can be documented with the enlargement of the optic cup. Capillary hemangioma of the retina appears as a yellow spot between a dilated tortuous feeding arteriole and a draining venule. Vision loss occurs due to exudation at the macular region. In the vitreoretinal form, epimacular membranes form, which causes macular traction detachment and decreased vision. Traction on the angiomas can lead to “free-floating” angiomas in the vitreous hemorrhage, and a combined traction rhegmatogenous retinal detachment can also occur. Vasoproliferative tumors of the retina are sporadic, and they appear as solitary pinkish-yellow raised vascular mass in the pre-equatorial retina, classically located inferiorly with minimally dilated feeder vessels, associated with intraretinal and extensive subretinal exudation and hemorrhage, secondary retinal detachment, premacular fibrosis, tractional RD, macular edema, RPE hyperplasia, and vitreous hemorrhage [10].

Prechiasmal Tumors

Primary tumors of the optic nerve and its sheath are frequently benign but cause vision loss with clinical neuropathy and variable proptosis as they progress. Intrinsic tumors such as gliomas cause visual loss by destroying the optic nerve fibers [12]. They can present in a variety of ways. The anterior presentation is characterized by signs of an anterior optic neuropathy with variably decreased visual acuity, depending on the mass's size and location, reduced color vision, a relative afferent pupillary defect, strabismus that usually is vertical, painless proptosis, and optic disc swelling. The posterior presentation presents similarly, except that the optical disc appears normal or pale [13,14].

Malignant astrocytomas produce a clinical course of rapidly progressive visual loss. A characteristic syndrome of the tumors originates in the proximal portion of the nerve. The initial symptom is monocular blurring vision. The fundus of the affected eye initially may appear normal but rapidly develop occlusive vascular disease involving the optic disc; there may be hemorrhage of the posterior pole, and neovascular glaucoma may develop. Within five to six weeks, both eyes become affected and completely blind. The malignant astrocytomas that originate in the distal position of the optic nerve produce a syndrome of progressive unilateral visual loss associated with a normal-appearing optic disc that eventually becomes pale. Optic nerve medulloepithelioma presentation depends on its location. When the tumor involves the orbital portion of the optic nerve, there is proptosis and optic disc swelling. More posterior lesions produce progressive retrobulbar optic neuropathy [15].

Hemangioblastoma of the optic nerve is a rare tumor; visual field defects on optic nerve hypoplasia, such as quadrantanopia, hemianopia, or arcuate, are a frequent initial presentation. Other features are proptosis and optic atrophy. Meningiomas and other extrinsic tumors produce visual decline via compression of the nerve or its vasculature [12]. The typical symptom of optic nerve meningioma (ONM) is gradual and slow, painless visual loss. There is no diagnostic visual field defect in ONM, but a wide variety of visual field defects can occur, including arcuate, altitudinal, paracentral, and cecocentral defects [16,17]. Visual acuity is variable, and some patients experience dyschromatopsia and short visual obscurations triggered by postural changes or head movements, which are always associated with swollen optic discs [17]. About one-third of patients with ONM have retinal-choroidal vessels that shunt venous blood from the retina to the choroid due to compression of the central retinal vein [14]. Schwannomas of the optic nerve are rare, and the clinical presentation is nonspecific but includes progressive visual loss associated with optic neuropathy and variable proptosis [15].

Chiasm

Structural lesions near the chiasm are pituitary adenomas (the most common cause of chiasmal vision loss in adults), meningiomas, gliomas, and aneurysms. In children, chiasmal hypothalamic gliomas and craniopharyngiomas are most commonly encountered. Intrasellar lesions initially cause supratemporal visual field defects, progressing clockwise in the right eye and anticlockwise in the left. Suprasellar lesions affect inferotemporal quadrants first. The prototypic visual field defect arising from the structural disruption of the optic chiasm is bitemporal hemianopia [18]. Chiasmal gliomas typically present with slow bilateral visual loss, optic-disc swelling or atrophy, strabismus, or bitemporal field defects [19]. Due to the proximity of sellar lesions to the optic nerves and chiasm, one of the most common and debilitating effects [20]. The classical presentation has bitemporal hemianopia or inferior quadrantanopia due to chiasmal compression and bilateral optic atrophy. Posterosuperior compression of the chiasm produces variable bitemporal hemianopia, usually beginning in the inferior temporal quadrant [12,19]. Quadrantanopia is generally of the inferior quadrant because of the superior location of the fibers [17]. Other visual field defects in chiasmal compression include unilateral defects and homonymous hemianopias. Unilateral defects can occur when the compression is at the cranial portion of the optic nerve. Homonymous hemianopias can occur if the chiasm is prefixed if only the lateral chiasm is involved, or if the lesion predominantly compresses the optic tract [21]. Retrochiasmal lesions between the optic chiasm and the visual cortex present with homonymous visual field defects and, at times, optic atrophy. Wedge-shaped homonymous hemianopias in the superior visual field (“pie-in-the-sky” defects) have been considered hallmarks of lesions of the Meyer loop in the anterior temporal lobe. Incongruous homonymous hemianopias have been linked to lesions of the optic tract, whereas congruous homonymous hemianopias have been linked to lesions of the retrogeniculate segment. Homonymous quadrantanopias, homonymous hemianopias with macular sparing, homonymous paracentral scotomas, temporal crescent-sparing homonymous hemianopias, and unilateral temporal crescent defects have been associated with lesions of the primary visual cortex [22].

Retrochiasmal Lesions

Retrochiasmal lesions between the optic chiasm and the visual cortex present with homonymous visual field defects and, at times, optic atrophy. Wedge-shaped homonymous hemianopias in the superior visual field (“pie-in-the-sky” defects) have been considered hallmarks of lesions of the Meyer loop in the anterior temporal lobe. Incongruous homonymous hemianopias have been linked to lesions of the optic tract, whereas congruous homonymous hemianopias have been linked to lesions of the retrogeniculate segment. Homonymous quadrantanopias, homonymous hemianopias with macular sparing, homonymous paracentral scotomas, temporal crescent-sparing homonymous hemianopias, and unilateral temporal crescent defects have been associated with lesions of the primary visual cortex [22].

Cerebral Cortex

Cortical blindness results from damage to the primary visual cortex or its immediate afferents. “Cortical blindness” includes left and right-sided hemianopias, superior and inferior homonymous quadrantanopias, and scotomas of the right and left visual hemifields. Most cases of cortical blindness are unilateral, but total vision loss can occur due to bilateral tumoral lesions of the retro chiasmal visual pathways. Cardinal features are complete loss of all visual sensation, including the perception of light and dark, loss of reflex lid closure to bright illumination/threatening gestures, retention of the pupillary light reaction and immediate response, the integrity of typical retinal structures, and regular extraocular movements [18,23].

Other Manifestations

Due to visual pathway lesions, palinopsia has an associated homonymous hemianopia visual field defect. In such cases, the lesion is presumed to spare the geniculocalcarine radiations. Most reported visual pathway lesions are right hemisphere lesions [24]. An afferent pupillary lesion refers to the retina, the optic nerve, the chiasm, the tract, and the optic radiations. An efferent pupillary lesion refers to lesions of parasympathetic and sympathetic innervation [25].

Optic pathway damage associated with radiosurgery

Since 1995, the research assessed by Leber has demonstrated that visual pathways are more susceptible to radiation harm than the other cranial nerves in the area [25]. They may be easier if, before radiosurgery, their function has been impaired by a tumor or prior surgery [26]. Ischemia and associated vascular damage in conventional radiotherapy have explained the possibility of optic atrophy due to damage to its axonal elements. In these cases, gliosis and demyelination may also be linked to neuropathy. Otherwise, the pathophysiological factors causing visual pathway damage in radiosurgery must be better understood. As a result, the definitions and cut-off points for radiation-induced optic neuropathy (RION) have yet to be well defined, and increased estimates of this diagnosis are typical in clinical practice [2]. In the literature, finding different ways of estimating it is expected. Some authors estimate it as a clinical decrease in visual acuity. In contrast, others use latency in evoked potentials as their primary study point because this is the first sign of RION.

When delineating the radiation field, the optic nerves, chiasm, and proximal tracts can be segmented independently or together. Because the anteroposterior extent of the chiasm and the contour of the visual pathway are variable, this delineation can be challenging. Coronal images are instrumental in defining the superior/inferior extent of the optic apparatus.

**Figure 2:**
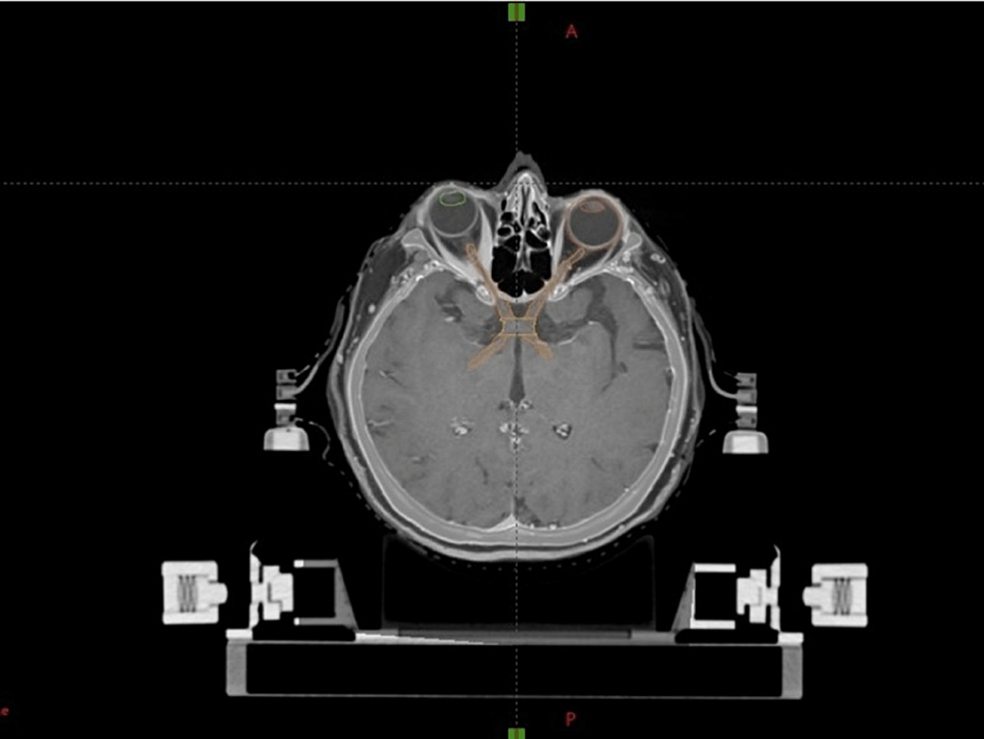
Co-registration of simulated tomography with magnetic resonance. Neutral skull position to observe the visual pathway in a single slice.

Potential sources of heterogeneity in this delineation that underlie dosimetric error are tumor compression of the visual pathway that makes boundary determination difficult, experience in segmenting the small volume that represents the optical apparatus, varying image quality due to magnetic resonance slice thickness, and rapid dose fall-off with highly conformal techniques (Figure 2).

When evaluating a patient with suspected RION, Milano et al. noted that most studies use the maximum point dose (maxD) across the optical apparatus as the dose metric to assess the risk of visual damage. Hence, this is the parameter to target. However, this dose may depend on the planning system and its metrics. They also explain that RION studies have characterized some factors that increase the risk of RION. Regarding re-irradiation, previous radiotherapy increases this entity’s possibility ten times. Accordingly, previous surgery could increase the susceptibility of the visual pathway to damage since surgical manipulation causes devascularization. Also, the pretreatment condition of the optic nerve, even in those patients who have not received previous radiation, is likely to affect post-treatment vision outcomes. Deng et al. demonstrated that the minimum dose causing optic neuropathy in cats is 12 Gy in non-compressed nerves and 11 Gy in compressed nerves. For this reason, it is crucial to perform a good evaluation of the visual status before planning the radiosurgery protocol. Other factors that may increase the risk of SRS toxicity include diabetes, hypertension, and collagen vascular disorders [27]. Overall, radiosurgical management is divided into two types. Those in which a single dose is used before the tumor and others in which hypofractionated doses are used. In the latter, the patient is seen on two to five consecutive days with distributed doses of radiation. Another essential aspect to consider is the radiosurgery modality. Hinikier et al. reported a dose model for hypofractionated radiosurgery. 

QUANTEC guidelines have recommended a maximum dose of ten to twelve Gy for a single-fraction treatment. In comparison, Stafford et al. from Mayo Clinic reported the safety of ten Gy as the median point maximum dose and less than 2% of RION with ≤12 Gy based on DVH-toxicity analysis. QUANTEC data estimates the risk of toxicity for optic nerve or chiasm maxD of 55 Gy at 3%, 55-60 Gy at 3%-7%, and 460 Gy at 47%-20%. Several studies have determined that a maximum dose limit in the optic apparatus associated with a reasonable risk of RION (1%) in patients without previous radiotherapy is 10 Gy in a single fraction, 20 Gy in three fractions, and 25 Gy in five fractions. John Adler explains that the unique radiation sensitivity of the typical optical apparatus precludes conventional radiosurgery when a lesion is within two millimeters of the anterior visual pathways. 

RION should be suspected in patients with decreased visual acuity and visual field defects after SRS. Still, it is necessary to rule out other more common diagnoses. RION is a diagnosis of exclusion. The incidence of RION following radiation treatment of head and neck or skull-base tumors ranges from 0% for <50 Gy to 16% for >70 Gy. Other entities should be taken into consideration before diagnosing RION [28]. RION is a complication that typically presents 10-20 months after treatment, with a mean of 18 months. However, this range is highly variable and can deliver from three months to eight years after treatment, so the history of SRS may be missed, especially in patients with a later presentation [29]. Since RION is a diagnosis of exclusion, other entities with progressive visual loss should be considered before diagnosing RION. In the case of a patient with low visual acuity after radiation exposure, common complications should be ruled out first. Ophthalmologic complications have been studied after using conventional radiotherapy in skull base tumors and ocular tumors; the complications that can occur by this type of radiation are severe dry eye, bullous keratopathy, corneal epitheliopathy, leukomas, and cataract [29]. Although SRS is relatively safe for ocular structures, ophthalmologic complications have been reported using SRS in regions close to the optic nerve [30]. An ophthalmologic slit-lamp examination should be performed considering possible diagnoses of anterior segment damage due to radiation. Radiation damage to the retina should also be considered, although this is less common than damage to the optic nerve [29]. Once common post-radiation exposure pathologies have been ruled out, other causes of visual acuity loss can be investigated. The differential diagnosis includes compression, infiltrative optic neuropathy, metastasis to meninges (meningeal carcinomatosis), or paraneoplastic optic neuropathy (Table 1) [31].

**Table 1:** Differential diagnosis of RION A-RION, anterior radiation-induced optic neuropathy; P-RION, posterior radiation-induced optic neuropathy; AION, anterior ischemic optic neuropathy; MRI-DTPA, magnetic resonance imaging - gadolinium DTPA; FA, fluorescein angiography; OCTA, optic coherence tomography angiography; ESR, erythrocyte sedimentation rate; CRP, C-reactive protein; CSF, cerebrospinal fluid; CGCA, giant cell arteritis

	Evolution of vision loss	Laterality	Pain	Visual acuity	Visual field abnormalities	Color vision deficits	Funduscopic exam	MRI findings	Other studies/tests	Visual prognosis
A-RION	Acute-subacute	Unilateral	Painless	Diminished	orbital, paranasal	Commonly abnormal	Optic disc swelling, microhemorrhages, microangiopathy, cotton wool spots	MRI not needed	Usually not needed	Usually poor
P-RION	Sudden, progressive over weeks	Unilateral (can become bilateral)	Painless	Diminished	Central defect	Commonly abnormal	Normal in the acute phase (pallor 6-8 weeks later)	Pre-contrast MRI (T1 and T2): normal MRI-DTPA: Optic nerve enhancement on T1	Early stage FA: retinal arterial narrowing, punctate and dispersed hemorrhages, retinal microaneurysms.	Poor risk of <20/200 vision. Potential for permanent blindness in both eyes
Tumor progression/recurrence	Usually progressive/variable (depends on the tumor)	Uni- or bilateral	Pain infrequent	Usually diminished/depends on the location	Arcuate, hemianopsia	Commonly abnormal	Normal or optic disc atrophy	Depends on the type of primary tumor	Depends on the type of primary tumor	Variable/usually poor if the tumor has a recurrence
Nonarteritic AION	Acute	Unilateral	Pain infrequent	Variable	Altitudinal defect	Commonly spares color vision	Disc edema, small cup-to-disc ratio	MRI not needed	OCTA might be useful in severity	Variable, 15% risk for the other eye within 5 years
Arthritic AION	Acute	Uni- or bilateral	Pain	Severe visual loss	Any defect (severe)	Commonly abnormal	DIisc edema, pallid retinal infarction	MRI not needed	ESR, CRP, and temporal artery biopsy (rule out CGA)	Usually poor, risk of the other eye within 10 days
Infiltrative optic neuropathies	Acute or subacute	Bilateral	Painless	Variable	Arcuate, hemianopic	Commonly abnormal	Edema, exudates, peripapillary hemorrhages	MR-DTPA: enlargement of the orbital and canalicular segments of the optic nerve	CSF: Malignant cells (>20mL of CSF needed)	Depends on the condition, usually poor
Paraneoplastic optic neuropathy	Progressive	Bilateral	Painless	Variable	Central defect	Commonly abnormal	Bilateral optic disk edema	T2 lesions and enhancement	PNMA2 (Ma2/Ta), anti-Yo, anti-Ma2 anti-CV2 antibodies	Poor and unpredictable

One of the main clinical features that can help distinguish between RION and other optic neuropathies is the onset of visual loss. In RION, visual loss usually occurs rapidly, within weeks to months [29,31]. The presentation of optic nerve compression due to a primary CNS tumor is usually more insidious, accompanied by other symptoms related to mass effect. Visual loss in RION is painless, so arteritic anterior ischemic optic neuropathy (AION) should be considered the first diagnosis in the presence of a painful acute decrease in visual acuity and alteration in visual fields. In non-arteritic AION, pain is a rare manifestation but may occur in 8% to 12% of patients, so it should be considered another option in these cases [32]. RION often presents unilaterally at onset, with the contralateral eye becoming affected over weeks. Infiltrative, paraneoplastic, and compression optic neuropathies usually present bilaterally but asymmetrically. The only pathology in the differential diagnosis generally unilateral during the disease is anterior radiation-induced optic neuropathy (A-RION) in the context of giant cell arteritis (GCA) [33]. The fundus in the earliest stages of posterior radiation-induced optic neuropathy (P-RION) is usually completely normal. It is until the sixth to eighth week that the edematous and atrophic optic disc and fundus pallor begin to be noticed [31,34]. In arteritic and non-arteritic AION, infiltrative optic neuropathies, and paraneoplastic syndromes, the fundus frequently changes from the beginning, presenting edema, microhemorrhages, and cotton-wool spots [33].

Optical coherence tomography angiography (OCTA) has proven effective in analyzing peripapillary vasculature in multiple sclerosis, glaucoma, and anterior ischemic optic neuropathy. It has also been demonstrated to individuate early vascular changes in radiation maculopathy, even in patients without ophthalmoscopic signs of the disease [35]. A lumbar puncture can be performed to evaluate for infiltrative neuropathies, where malignant cells would be found [36]. In case of suspicion of paraneoplastic syndrome, serology may be helpful by asking for PNMA2 (Ma2/Ta), anti-Yo, anti-Ma2, and anti-CV2 antibodies [37]. There are considerations when ordering MRI in RION; early MRI cannot differentiate RION from a recurrent tumor. And the other is that the pathology may be missed, as the image through the optic chiasm may be limited to one slice. Therefore, it is essential to specify that small 3T slices (3 mm or less) are needed [38].

Ophthalmologic evaluation after stereotactic radiosurgery

A patient undergoing SRS must undergo an ophthalmologic assessment before and after the procedure because radiation used in this type of treatment may trigger damage to the visual pathway through multiple points, including the lacrimal glands, lens, retina, optic nerve, optic chiasm, optic tracts, or occipital cortex. Because radiation-related vision loss is multifactorial, it is imperative to have a baseline ophthalmologic examination. Comorbid conditions can contribute to RION. For patient follow-up and visual prognosis, it is essential to document the presence of diabetic retinopathy, hypotensive retinopathy, or glaucomatous optic neuropathy [2]. Multiple endpoints can be used to assess visual impairment, including symptoms, visual acuity, visual field limitations, optic nerve examination, and even studies such as ash, fluorescein angiogram (FAG), or MRI. The patient's symptoms and time of evolution are essential in the follow-up since one of the main characteristics of RION is the loss of non-painful vision [31]. Other symptoms reported by the patient are necessary to record, such as paracentral scotomas or hemianopsia. In the fundus examination, the most relevant data is the presence or absence of retinopathy, exudates, hemorrhage, and papilledema because if we find them, these would be more suggestive of other pathologies and not of RION. It is essential to consider that the fundus may appear practically normal in RION in acute phases but may change to pallor in six to eight weeks [34]. Although there is no clear recommendation, the patient needs an automated visual field examination, as it is a more objective parameter for visual defects secondary to optic neuropathy. Fluorescein angiography is a study that provides little data in cases of RION and is impractical to perform routinely, so its usefulness is reduced to diagnosing other pathologies. 

Optic nerve optical coherence tomography (OCT) can be a critical study in the baseline assessment of the patient undergoing SRS. Measurements of the retinal nerve fiber layer can be an objective measure of nerve inflammation or atrophy. OCT can help detect early axonal damage and predict the visual outcome by analyzing the ganglion cell complex. It can help diagnose and follow compressive optic nerve and chiasmatic diseases [39]. Since there is significant variability in the findings of each test or parameter, it is preferable to use standardized scales that can more objectively state the patient's visual status [2]. The primary scales used to evaluate organ-specific toxicity due to radiation treatment are The Radiation Therapy Oncology Group/European Organization for Research and Treatment of Cancer, Late Effects in Normal Tissue/Subjective, Objective, Management, Analytic (RTOG/EORTC, LENT-SOMA) scale, published in 1995, and the Common Terminology Criteria for Adverse Events (CTCAE) Version 5 scale [40]. The parameters evaluated by the RTOG/EORTC, LENT-SOMA scale are divided into subjective data (visual symptoms, pain, sensitivity to light), objective data (visual acuity, visualization of the optic nerve, retina, etc.), medical management (use of analgesics, lubricants, etc.) and analytical data (slit-lamp examination, MRI, FAG, etc.). The CTCAE version 5 scale evaluates blurred vision, cataracts, corneal ulcers, dry eye, papilledema, retinopathy, and others, but only grades optic nerve toxicity based on visual acuity and does not incorporate visual field loss. It is recommended to use both scales in patients with suspected RION [40].

Although there has yet to be a date recommendation on what essential studies or assessments an ophthalmologist should perform before a patient undergoes SRS. Ideally, baseline visual acuity, automated visual fields, optic nerve OCT and retinal ganglion cell layer should be recorded. The periodicity of assessment will depend on the criteria of the ophthalmologist, ideally with more periodic visits in patients with an accumulated radiation dose of more than 50 Gy. RION is a complication that can appear many years after radiation exposure, so the patient should be under ophthalmologic follow-up.

Therapeutics

Currently, there is no universally accepted management to treat RION; to our knowledge, no randomized clinical trial has been performed to evaluate which therapy is best to treat RION, and due to RION being a relatively rare complication, articles concerning RION treatment include few patients. Due to there being no consensus on treatment, therapeutic measures used to treat radiation brain necrosis (RBN) have been extrapolated to treat RION, including the use of intravenous corticosteroids at high doses, anticoagulants, hyperbaric oxygen (HBO), and recently, the alternative of using ramipril (ACE inhibitor) and antiangiogenic therapies [41].

Corticosteroids

Corticosteroids have been frequently used to treat RION to reduce edema and demyelination, although with limited success rates. When treating RBN with steroids, dexamethasone 4-10 mg orally or intravenously four times daily with tapering doses of 2-4 mg every five to seven days is generally used. Alternatively, one may enlist high-dose intravenous methylprednisolone at 1 g/day for five days, followed by oral methylprednisolone at 80 mg/day for one week, followed by a several-week taper. Using steroids in RION has yet to be reported to be very effective. Girkin et al. reported four patients with RION, all receiving systemic corticosteroids and one receiving adjuvant HBO therapy [42]. One of the patients who received corticosteroids alone had acuity improved from 20/70 to 20/30. Lee et al. reported three cases of RION, and one stabilized on corticosteroids alone [43]. In addition to being a therapy that often does not work, the adverse effects of using high corticosteroids should be considered.

Anticoagulants

Since they have been shown to improve blood flow to irradiated tissue and prevent and repair small vessel endothelial damage, anticoagulants, primarily warfarin, and heparin are often prescribed [44]. However, this therapy has not significantly improved neurological complications from radiation. Therefore, this effect is more conceptual than practical. Anticoagulant use has even been linked to the development of RION in some case reports, leading experts to believe that these patients would not benefit significantly from this course of treatment [45].

ACE Inhibitor (Ramipril)

The blood-brain barrier-crossing ramipril is thought to be able to prevent radiation-induced axonal damage by lowering pro-inflammatory cytokines. Ramipril was tested in rats exposed to a dose of 30 Gy and treated early with this drug. Compared to untreated rats, which developed optic nerve demyelination, there was a protective effect on the development of RION. However, an established standardized dose in humans has yet to be documented [46,47].

Hyperbaric Oxygen

HBO is one of RION's most widely used and aggressive therapies. This therapy is thought to mitigate the effects of radionecrosis by artificially increasing oxygen tension in damaged tissues, promoting angiogenesis, and causing persistent reoxygenation of previously hypoxic tissues [48,49]. This therapy successfully treats radiation damage to soft tissues and bone, but its efficacy in treating nervous system damage has yet to be proven [50]. One study, including 13 patients with RION, treated more than two weeks after onset of vision loss with HBO (2.0 atm; and in 11 cases with concurrent systemic corticosteroids), showed no visual improvement. However, HBO may be successful when using at least 2.4 atm for patients where symptoms have begun recently, and optic pallor has not yet developed [41]. Despite having little benefit, some authors recommend giving HBO as soon as possible after the onset of visual loss [41,51]. Current guidelines suggest a protocol of 30 sessions at a pressure of 2.4 ATA (atmospheres absolute). One advantage of this therapy is that it is a relatively safe procedure. However, there may be a regression in visual acuity after treatment discontinuation [51,52].

Bevacizumab

A double-masked, randomized clinical trial evaluating the use of systemic bevacizumab in RBN secondary to radiation from head and neck or brain cancers revealed Class I efficacy with a symptomatic improvement of neurological deficits and radiographic reduction of CNS necrosis volume as measured by MRI [53]. A case report on the systemic use of bevacizumab in RION describes improvement in a patient treated with intravenous bevacizumab, dexamethasone, and pentoxifylline. After three years, the patient improved visual acuity to 20/25 OS and 20/20 OD, with intact color vision bilaterally, from 20/100 OS and no light perception (NLP) OD [54].

A clinical case series involving 14 patients evaluating intravitreal bevacizumab for radiation optic neuropathy related to plaque radiotherapy revealed enhanced optic disc edema and hemorrhage and improved visual acuity in half of the patients [54]. Considering that there is currently no proven therapy for RION, these studies and reports on the use of antiangiogenics are of great interest, even though there are no randomized clinical trials of anti-VEGF use to treat RION.

## Conclusions

Radiation dose, dose fraction, previous treatment, and adjuvant chemotherapy are the factors most associated with the development of RION. In a patient with a visual deficit after radiosurgery, the growth of the lesion is the principal diagnosis to consider. Its ophthalmologic manifestations are typically progressive and insidious. Adjacent edema, radiation-induced neoplasms, arachnoid adhesions around the optic chiasm, radiation retinopathy, and RION are the differential diagnoses related to this treatment modality's structural and biochemical changes. The existence of patient-specific factors to predict the likelihood of RION is still controversial. Although there are no standardized protocols for its treatment, early intervention has been shown to be associated with less catastrophic outcomes. Knowing the clinical presentation of these entities and the behavior to follow in the event of suspicion maximizes the scope of radioneurosurgery by reducing its risks.
